# Evaluating sugar-sweetened beverage tax effects: online price and sales data from grocers in Canada

**DOI:** 10.1093/heapro/daaf203

**Published:** 2025-12-02

**Authors:** Rachel Prowse, Daniel Zaltz, Kayla Crichton, Kierra Dooley, David Hammond, Yanqing Yi, Marie-Claude Paquette, Peizhong Peter Wang, Kim Raine, Scott V Harding

**Affiliations:** Population Health and Applied Health Sciences, Faculty of Medicine, Memorial University, Health Sciences Centre, 300 Prince Philip Drive, St. John's, NL, Canada, A1B 3V6; Department of Nutritional Sciences, University of Toronto Temerty Faculty of Medicine, Medical Sciences Building, 1 King's College Cir Suite 5253A, Toronto, ON, Canada, M5S 3K3; Department of Biochemistry, Faculty of Science, Memorial University, 45 Arctic Avenue, St. John's, NL, Canada, A1C 5S7; Department of Biochemistry, Faculty of Science, Memorial University, 45 Arctic Avenue, St. John's, NL, Canada, A1C 5S7; School of Public Health Sciences, University of Waterloo, 200 University Ave West, Waterloo, ON, Canada, N2L 3G1; Population Health and Applied Health Sciences, Faculty of Medicine, Memorial University, Health Sciences Centre, 300 Prince Philip Drive, St. John's, NL, Canada, A1B 3V6; Institut national de santé publique du Québec, 190, boulevard Crémazie Est, Montréal, QC, Canada, H2P 1E2; Population Health and Applied Health Sciences, Faculty of Medicine, Memorial University, Health Sciences Centre, 300 Prince Philip Drive, St. John's, NL, Canada, A1B 3V6; Centre for New Immigrant Well-Being (CNIW), 96 Scarsdale Road, Toronto, ON,NT-TESTCanada, M3B 2R7; Dalla Lana School of Public Health, University of Toronto, 155 College Street, Room 534, Toronto, ON,NT-TEST Canada, M5T 3M7; School of Public Health, University of Alberta, 11405 87 Ave NW, Edmonton, AB, Canada T6G 1C9; Department of Biochemistry, Faculty of Science, Memorial University, 45 Arctic Avenue, St. John's, NL, Canada, A1C 5S7

**Keywords:** sugar, observational study, intervention, health promoting policies, healthy public policy

## Abstract

Newfoundland and Labrador (NL) introduced Canada’s first sugar-sweetened beverage (SSB) tax in September 2022. Compared with national averages, NL has higher intakes of SSBs, lower intakes of plain water and milk, with higher rates of overweight and obesity and diabetes. Taxing SSBs is a recommended intervention but real-world effectiveness of SSB taxes requires more investigation. We evaluated changes in weekly beverage prices and sales pre- and post-tax implementation, comparing NL (intervention) to non-tax regions in Canada (control). We used a controlled interrupted time series to evaluate prices from grocery store websites 3 months pre- and post-tax. We observed no differences-in-differences in the intercept [*β* = −0.024, 95% confidence interval (CI) −0.15–0.10, *P* = .70] or slope (*β* = 0.00, 95% CI −0.02–0.02, *P* = .99) of price changes. We used a repeat cross-sectional study to compare total annual sales of beverage categories in the year pre- and post-tax. Per capita sales in litres of taxable SSB decreased more in NL (−11.6%) than non-tax regions (−6.7%). Per capita sales of diet beverages (+4.4%) and unsweetened water (+2.2%) increased in NL. The NL SSB tax had no immediate impact on retail prices of taxable SSBs measured on product selection pages on grocery websites. Beverage purchasing shifted in NL since the SSB tax start date, however, it is difficult to isolate the impact of the SSB tax from broader market trends or other influencing factors. Long-term evaluation of the NL SSB tax is needed.

Contribution to Health Promotion

- This study shows real-life impacts of Canada’s first sugar-sweetened beverage (SSB) tax on beverage prices and sales.
- Retailer sales data from almost 600 stores showed that sales of taxable beverages decreased slightly more in Newfoundland and Labrador with an SSB tax than in comparison provinces with no tax.
- Prices for taxable beverages on grocers’ webpages did not differ between regions, meaning the SSB tax was not clearly communicated to consumers.
- Clear communication of an SSB tax to consumers is not guaranteed. Real-world evidence is needed to evaluate how implementation tempers effectiveness of SSB taxes as a public health intervention.

## INTRODUCTION

Approximately 40% of all Canadians’ free sugar intake comes from sugar-sweetened beverages (SSBs) (Rana *et al*. 2021). Currently, over 80% of Canadian youth aged 9–18 consume more than the recommended intake of free sugars (Rana *et al*. 2021). High intake of SSBs is associated with numerous health risks, including central adiposity (Nguyen *et al*. 2023), elevated blood lipids (Haslam *et al*. 2020), insulin resistance (Ma *et al*. 2016), diabetes (Malik and Hu 2012), coronary heart disease (Huang *et al*. 2014), and dental caries (Valenzuela *et al*. 2021). SSB consumption is linked to at least 12 types of cancers, both independently and through obesity-mediated pathways (Lieffers *et al*. 2018). Excessive SSB consumption contributes significantly to direct and indirect healthcare costs in Canada, totalling $830 million, further exacerbated by inadequate milk consumption ($666 million) (Lieffers *et al*. 2018).

More than 100 countries have implemented SSB taxes, which can serve as effective financial disincentives and revenue generators, encouraging shifts in consumer behaviour and reinvestment of tax dollars for social welfare (World Bank 2020, World Health Organization 2023). An SSB tax is expected to reduce disease risk by increasing SSB prices leading to reduced SSB demand and consumption, sugar and calorie intake, improved energy balance, and reduced rates of obesity (Claudy *et al*. 2021, Andreyeva *et al*. 2022). A well-designed and effective tax can serve as a signal to both consumers (warning consumers of risks) and industry (encouraging product reformulation) (Le Bodo *et al.* 2016). Modelling studies have shown that SSB taxes can reduce the prevalence of overweight and obesity, with a 20% tax estimated to decrease obesity by 1.3% in the UK (180 000 people), 6.7% in Brazil (2.8 million people), and similar reductions observed in middle-income countries like Mexico and South Africa (Briggs *et al*. 2013, Nakhimovsky *et al*. 2016, Basto-Abreu *et al*. 2024). Implementing a national SSB tax was estimated to prevent 12 053–21 777 new cases of obesity-related cancer in Canada over 25 years (Jones *et al*. 2017). The real-world effectiveness of these taxes still requires more investigation (Popkin and Ng 2022).

Meta-analytic evidence has shown SSB taxes to be associated with increased prices of taxed beverages along with lower sales, suggesting that these interventions are effective in altering consumer behaviour in relation to beverage purchasing (Andreyeva *et al*. 2022). The impact of an SSB tax often depends on how much of the cost of the tax is passed on to consumers in the form of raised beverage prices, and this rate can vary from no cost passed through to values over 100% of the cost of the tax (Andreyeva *et al*. 2022). Despite the fact that a number of previously implemented SSB taxes raise beverage prices only a relatively small amount (10% or less), an average reduction in sales of taxed beverages of ∼15% was observed (Andreyeva *et al*. 2022). One study examining the SSB tax in Berkeley, California found an average price increase of up to 4%, while consumption at the chain supermarket studied declined by 7%–12% (Bollinger and Sexton 2023). After SSB taxes were implemented in Oakland and San Francisco, California, a 14% and 15% increase in total SSB retail price were observed, respectively (Falbe *et al*. 2020). One study of the Philadelphia SSB tax found a 34% price increase along with a 46% reduction in quantity of taxed beverages purchased in the city (Seiler *et al*. 2020), while another found an overall reduction of 38% in total volume sales (Roberto *et al*. 2019).

Newfoundland and Labrador (NL) became the first Canadian province to introduce an SSB tax on 1 September 2022 (Government of NL 2022). This initiative targets a province with some of the worst dietary and health outcomes in Canada. NL residents consume 15% more SSBs than the national average, coupled with the lowest intakes of water and milk (Jones *et al*. 2019). Furthermore, NL has the highest combined prevalence of overweight and obesity, diabetes, and all cancers, with a particular burden from colorectal cancer (Brenner *et al*. 2020, Statistics Canada 2023). The tax was framed as a health-focused initiative to promote better beverage choices and thus improve health outcomes in the province (Government of NL 2021). The provincial excise tax of $0.20/l applies to all ready-to-drink SSBs (and concentrated drink products) with the exception of chocolate milk and 100% fruit juice; it is imposed on SSB wholesalers and is intended to be passed through from wholesalers to retailers, who receive no remittance with the expectation of passing the tax on to the consumer (Government of NL 2022). However, little guidance on implementation is provided to retailers, with no requirements on where or how the tax is to be displayed to the consumer—this appears to be at the retailer’s discretion (Government of NL 2022). In order to properly evaluate the policy, it is vital to explore how retailers chosen to communicate the tax to consumers, and if this is reflected in the form of increased SSB prices.

While modelling studies predict positive outcomes, the true impact of NL’s SSB tax remains unknown. This project is the first study to evaluate changes in beverage prices and sales in NL before and after the introduction of the NL SSB tax, compared with non-tax regions.

### Research objectives and hypotheses

We aimed to measure changes in (i) retail beverage prices 3 months before and after the tax using data from online grocery websites, and (ii) beverage sales (revenue) in grocery stores, mass merchandizers, and drug stores. We hypothesized that the NL SSB tax would immediately increase prices of SSBs subject to the tax in NL, and these prices would remain higher than those in non-tax regions during the 3-month post-tax period. We did not generate specific hypotheses for beverage sales due to aggregated data limitations but anticipated differences in sales for taxable and non-taxable beverages across study periods and regions, consistent with the effects of an SSB tax. Hypotheses and analyses plans were not formally pre-registered.

## MATERIALS AND METHODS

This project is a natural experiment with the NL SSB tax as the intervention, using non-tax regions as controls. Two studies are included to evaluate the changes in beverage prices and sales before and after the tax. No ethical approval was needed as there was no human data collected. Hypotheses and analytic plans were generated before the data were collected.

### Study A: changes in beverage prices on online grocery websites

#### Research design

To evaluate changes in beverage prices, we used a controlled interrupted time series measuring online prices of beverages from grocery store websites.

#### Sampling

We collected a clustered random sample of national grocery retailers with online platforms present in NL and non-tax regions [Nova Scotia (NS), New Brunswick (NB), Manitoba (MB), and Yukon Territory (YT)]. These provinces were chosen because NL shares a grocery market with other Atlantic provinces (NS and NB) allowing for comparison within the same regional market; Manitoba has similar SSB intakes (Jones *et al*. 2019) and similar income levels (Government of Canada 2022) to NL and is generally considered a ‘have-not’ province (Cooke 2023) which allows for comparison to a similar economic and health context but different geographic context and market; Yukon is a Northern Territory in Canada which acts as a comparison for remote stores with online platforms located in Northern NL and was the only territory with at least two of the three grocery chains. From a list of 178 grocery store locations stratified by owner [Walmart, Sobeys, and Loblaws (Dominion in NL, Atlantic Superstore in NS and NB, and Real Canadian Superstore in MB and YT)], we randomly selected one store from each chain. During pilot testing of online pricing, we determined that beverage prices did not differ within a region within a retailer on the same day (i.e. beverage prices in all Sobeys in NS were identical). Thus, we did not collect data for more than one store type per region with the exception of Labrador City, NL which we added to our store sample due to government interest in the only online grocer available in Labrador. Sobeys retailers are not located in YT, so only Walmart and Loblaws stores were included. A total of 15 store locations were audited (NL: *n* = 4, NS: *n* = 3, NB: *n* = 3, MB: *n* = 3, YT: *n* = 2).

#### Data collection

Trained research assistants used an adapted version of the Beverage Tax Food Store Observation Form (Li *et al*. 2018) to collect online beverage prices. During pilot testing, the tool was adapted for Canada where research assistants recorded every ready-to-drink beverage and purchase unit size available for sale in two grocery stores, one drug store, and one convenience store in St. John’s, NL, Canada. We also identified the top purchased beverages from Coca-Cola (Said 2013a) and Pepsi (Said 2013b), as well as top selling energy drinks (Institut national de la santé publique du Québec 2010). From these activities, we adapted the tool for NL to monitor the prices of 53 unique beverages across 8 categories: soft drinks, sports drinks, energy drinks, juice and fruit drinks, iced teas/lemonades, bottled coffee drinks, waters, and milk and soy beverages (see Supplementary File 1). We included SSB types that would be taxed (e.g. regular soft drinks, energy drinks, fruit drinks) and other beverages (i.e. sweetened with non-nutritive sweeteners, sugary, and plain) for which the tax would not be applied (e.g. diet soft drinks, juice, chocolate milk, regular milk) (Government of NL 2022). Specific beverage brands were specified and were not replaced with alternative brands to reduce brand-specific impacts on price, with the exception of milk where we used a region-specific milk brand to reflect the local market.

Research assistants visited store websites for each chain, entered the selected store’s address, and searched for each beverage type. Regular and sale prices of all purchase units posted online were recorded, even if the purchase unit was sold out. After 1 September 2022, research assistants were instructed to look for the value of the SSB tax on the product selection page (i.e. where a customer can add a product to their cart) and recorded it for any beverage types, if listed.

Data were collected intermittently throughout each week of the pre- and post-intervention study period for 3 months prior to the implementation of the NL SSB tax (June–August 2022) and for the 3 months after implementation (September–November 2022). Data was verified for accuracy by reviewing regular and sale prices to confirm that sale prices were cheaper per purchase unit, and reviewing that sale prices represented cost per purchase unit (i.e. 12 × 355 ml) and not cost per item (e.g. 355 ml) nor total cost for all purchase units when there was a reduce price per quantity sold sale (e.g. $10 when you buy two). Sale prices for 155 purchase units were recalculated when the cost per item was misreported as the combined cost for multiple purchase units rather than the cost per single purchase unit. All prices were reviewed by beverage types, sizes, and stores for accuracy and plausibility; no data were deemed inaccurate or implausible and all data were retained.

#### Statistical analysis

We visually assessed the distributions of data collected throughout the study period using scatterplots and smoothed lines. We estimated the change (step) and trend (slope) of retail price per 100 ml of taxed SSBs in NL compared with the SSBs in non-taxed locations using a controlled interrupted times series analysis (Lopez Bernal *et al*. 2017). We defined NL as the intervention group, all other locations combined as the control group, 1 September 2022 as the intervention, and a variety of potential confounders identified via causal diagrams. To our knowledge, there were no other known major changes to food policies in intervention or control groups beyond the SSB tax. We adjusted our estimates for potential confounders including overall economic output, inflation, local food environment, geography, store ownership, and bulk purchase units. We identified the following time-fixed confounders: if the product was sold as single items or as multiples (e.g. 12-pack cans), corporate ownership of the product’s store (as parent company may impact prices more than individual store locations), region size (rural, small, medium, large) (Statistics Canada 2013), and the Modified Food Retail Index (ordinal, 0–5) (Statistics Canada 2022). The Modified Food Retail Index (measured at one time point but disaggregated to census dissemination area in which the store was located) is provided by Statistics Canada (Accessed February 4, 2025) and reflects the proportion of food retailers that sell a wide selection of fresh foods out of all food retailers within a 1-km network buffer. We identified the following time-varying (monthly) confounders: total worker compensation (Statistics Canada 2024b) as a proxy for overall economic growth in each province, and the consumer price index for all foods and beverages at the national level ( Statistics Canada, Accessed February 4, 2025) as a measure of inflationary changes.

We clustered data per week (mean weekly price) and estimated autoregressive moving-average models with generalized least squares (Hansen 2007, Bhaskaran *et al*. 2013). We assessed autocorrelation visually and with partial autocorrelation functions, and determined autoregressive terms by refitting many models and selecting the best one using a combination of fit statistics and visual inspection of model residuals (Lopez Bernal *et al*. 2018). Our dependent variable was price per 100 ml for taxable SSBs, and our target parameters were (i) the policy × group interaction (difference in the difference of average price immediately following the tax, comparing intervention to control) and (ii) the group × post-policy time interaction (difference in the difference of the average price change per week during the 3-months following the tax, comparing intervention to control). We report estimates, 95% confidence intervals (CIs), and *P*-values. We conducted all analyses using R version 4.2.2 (R Foundation for Statistical Computing, Vienna, Austria) with a significance threshold of *α* = 0.05.

### Study B: changes in beverage sales in grocery stores, mass merchandizers, and drug stores

#### Research design

To evaluate changes in beverage sales, we used a repeat cross-sectional design to compare total annual sales of ten beverage categories before and after the SSB tax implementation date in NL versus the Maritime provinces [NS, NB, Prince Edward Island (PEI), henceforth referred to as Martime provinces] where there is no SSB tax (Table 1). MAR is the most appropriate comparison market to NL reducing the impact on region-specific impacts on revenue that might exist across Canada.

**Table 1.** Sample characteristics of online retail beverage prices before and after implementation of the NL SSB tax.

Characteristic	Overall (*N* = 13 167)^a^	NL (*N* = 3197)^a^	NB (*N* = 2783)^a^	NS (*N* = 2713)^a^	MB (*N* = 3003)^a^	YT (*N* = 1471)^a^
Study period						
Pre-tax	7463 (57%)	1818 (57%)	1625 (58%)	1499 (55%)	1676 (56%)	845 (57%)
Post-tax (3 month follow-up)	5704 (43%)	1379 (43%)	1158 (42%)	1214 (45%)	1327 (44%)	626 (43%)
Region size^b^						
Rural (<1000)	2038 (15%)	0 (0%)	1087 (39%)	0 (0%)	951 (32%)	0 (0%)
Small pop. centre (1000–29 999)	6303 (48%)	2290 (72%)	842 (30%)	1700 (63%)	0 (0%)	1471 (100%)
Medium pop. centre (30 000–99 999)	854 (6.5%)	0 (0%)	854 (31%)	0 (0%)	0 (0%)	0 (0%)
Large urban pop. centre (100 000+)	3972 (30%)	907 (28%)	0 (0%)	1013 (37%)	2052 (68%)	0 (0%)
Modified food retail environment index^c^						
0	7741 (59%)	1492 (47%)	1941 (70%)	1721 (63%)	2001 (67%)	586 (40%)
1	2590 (20%)	1705 (53%)	0 (0%)	0 (0%)	0 (0%)	885 (60%)
2	1994 (15%)	0 (0%)	0 (0%)	992 (37%)	1002 (33%)	0 (0%)
3	0 (0%)	0 (0%)	0 (0%)	0 (0%)	0 (0%)	0 (0%)
4	842 (6.4%)	0 (0%)	842 (30%)	0 (0%)	0 (0%)	0 (0%)
Retail store ownership						
Loblaws	4517 (34%)	806 (25%)	854 (31%)	992 (37%)	980 (33%)	885 (60%)
Sobeys	3999 (30%)	899 (28%)	1087 (39%)	1013 (37%)	1000 (33%)	0 (0%)
Walmart	4651 (35%)	1492 (47%)	842 (30%)	708 (26%)	1023 (34%)	586 (40%)
Beverage category^d^						
SSBs, subject to NL tax	7531 (57%)	1792 (56%)	1538 (55%)	1570 (58%)	1773 (59%)	858 (58%)
SSBs, exempt from NL tax	196 (1.5%)	61 (1.9%)	83 (3.0%)	33 (1.2%)	12 (0.4%)	7 (0.5%)
Unsweetened beverages	2184 (17%)	495 (15%)	481 (17%)	431 (16%)	529 (18%)	248 (17%)
Diet beverages	3256 (25%)	849 (27%)	681 (24%)	679 (25%)	689 (23%)	358 (24%)
Package type						
Single unit	6860 (52%)	1728 (54%)	1480 (53%)	1411 (52%)	1474 (49%)	767 (52%)
Multiple unit package	6306 (48%)	1468 (46%)	1303 (47%)	1302 (48%)	1529 (51%)	704 (48%)

^a^ *n* (%).

^b^Region size from Canada Census 2021.

^c^Density measure of distance to food outlets that carry fresh fruits and vegetables and other healthy staples, derived from the Canadian Food Environment Dataset using the 1 km network buffer data (0 = no outlets, 1 = lowest density, 4 = highest density).

^d^See Supplemental File 1 for beverages included by category.

#### Sampling

Data from the grocery banner, drug retailers, and mass merchandizers channel were purchased from ACNielsen Company of Canada (ACNielsen), including a total of 129 stores (130 stores post-tax) in NL and 390 stores (389 stores post-tax) in MAR. The channel included stores-owned by Loblaws (NL: *n* = 47, MAR: *n* = 129 pre-tax, *n* = 130 post-tax), Sobeys (NL: *n* = 43; MAR: *n* = 185), and Walmart (NL: *n* = 11; MAR: *n* = 33). The NL market also included 14 stores from a provincial grocery chain (*n* = 15 post-tax).

#### Data collection

The sales data were sourced from point-of-sale scanners in stores. All sales made at stores are captured by ACNielsen; our data included only a subset of sales from nine ready-to-drink beverage type [soft drinks, juices and drinks (shelf-stable, and refrigerated), energy drinks, iced teas, coffee-type drinks, waters (flat and carbonated), milk]. These categories were industry-defined and included a variety of beverage types that were sugar-sweetened, sweetened with non-nutritive sweeteners, and unsweetened which have varied subjectivity to the NL SSB tax. Lists of beverage products within each category were provided to the research team from ACNielsen which we coded as ‘sugar-containing’ (free or added) or ‘no sugar’ using product name, type, and nutrient content and ingredient lists obtained from product websites when necessary. Products were also coded as ‘taxable’ or ‘non-taxable’, as per the NL SSB tax regulations (Government of NL 2022); see Supplemental File 2.

Total sales in dollars and units (L) were provided by beverage category, reported by ‘sugar-containing’ vs ‘non-sugar-containing, and ‘taxable’ vs ‘non-taxable’ for two markets [NL (intervention) vs MAR (control)] for the year before (4 September 2021–3 September 2022) and after (3 September 2022–2 September 2023) the tax was implemented in NL. The average price per unit by beverage category was provided, calculated by the total sales in dollars divided by the total sales in volume.

#### Data analysis

We derived beverage groups relevant to the NL SSB tax by combining total sales data from multiple beverage categories and sub-categories:

1. SSBs, taxable: sugar-containing soft drinks, energy drinks, iced teas, coffee-type drinks, waters, taxable milk (all sugar-containing flavoured milk except chocolate), and taxable juice and drinks (all sugar-containing juices and drinks, except 100% juices)
2. Diet beverages: non-sugar-containing, sweetened with non-nutritive sweetener, soft drinks, energy drinks, iced tea, coffee-type beverages, juices/drinks
3. Chocolate milk: sugar-containing chocolate-flavoured milk, excluding shake-type milks
4. 100% juice: fruit and vegetable juices that is labelled as, or contains only, 100% juice (including from concentrate).
5. Plain milk: unflavoured milk with no added sugar.
6. Water: flavoured or unflavoured, flat or carbonated water with no added sugar.

Data were standardized per 100 000 people from Statistics Canada’s monthly estimates in NL [September 2021–August 2022 (pre-tax) *n* = 529 536; September 2022–August 2023 (post-tax) *n* =535 826] and MAR (pre-tax *n* = 1 980 994; post-tax *n* = 2 041 760) (Statistics Canada 2024a). We used the mean Consumer Price Index for food (Statistics Canada n.d.) across study periods in NL and MAR to adjust the average price per litre of beverages in each location during the pre-tax period to account for the impact of inflation on food prices. No statistical analyses were possible due to the aggregate values by beverage category provided by ACNielsen. We calculated the absolute difference (post-tax minus pre-tax) and per cent change in each region for our derived beverage groups and all individual beverage types according to sugar-containing and taxable statuses.

## RESULTS

### Beverage prices

We collected complete data on retail prices from 7531 beverages that would be taxable under the NL SSB tax, of which 24% (*n* = 1792) came from grocers in NL and the remaining 74% (*n* = 5739) from non-tax regions (Table 1). The mean pre-tax price/100 ml of SSBs did not differ between NL and non-tax regions ($0.30 vs $0.31, 95% CI of mean difference −0.006–0.031, *P*-for-difference = .19) (Table 2). We observed parallel trends in prices of SSBs during the pre-tax period across intervention and control locations, and no differences-in-differences in the intercept (*β* = −0.024, 95% CI −0.15–0.10, *P* = .70) or slope (*β* = 0.00, 95% CI −0.02–0.02, *P* = .98) of price changes in response to the new tax. These results were consistent within unadjusted models that omitted control locations (Table 2). There was no evidence of a price change within the 3 months following the implementation of the SSB tax.

**Table 2.** Unadjusted and adjusted differences in the price per 100 ml of SSBs, subject to the NL tax (taxable^a^) before and after implementation of the NL SSB tax.

	Unadjusted, no control provinces		Unadjusted, control provinces		Adjusted, control provinces	
	*β* (95% CI)	*P*-value	*β* (95% CI)	*P*-value	*β* (95% CI)	*P*-value
Policy × group			−0.01 (−0.14, 0.11)	.80	−0.02 (−0.15, 0.10)	.70
Post-intervention time × group			0.002 (−0.02, 0.02)	.87	0.00 (−0.02, 0.02)	.98
Intercept	0.30 (0.22, 0.38)	<.001	0.29 (0.25, 0.34)	<.001	0.65 (−3.77, 5.08)	.77
Pre-intervention time (week)	0.002 (−0.01, 0.01)	.76	0.001 (−0.006, 0.008)	.79	0.002 (−0.01, 0.01)	.73
Policy	−0.011 (−0.13, 0.11)	.86	−0.008 (−0.07, 0.05)	.80	−0.01 (−0.07, 0.05)	.74
Post-intervention time (week)	−0.002 (−0.02, 0.01)	.84	0.00 (−0.01, 0.01)	.97	0.00 (−0.01, 0.01)	.99
NL (ref: control provinces)			0.02 (−0.06, 0.10)	.63	0.02 (−0.06, 0.10)	.64
Pre-intervention time × group			−0.001 (−0.01, 0.01)	.92	0.001 (−0.01, 0.02)	.88
Total worker compensation					0.00 (0.00, 0.00)	.75
Consumer price index, all foods, national					−0.002 (−0.03, 0.02)	.89
Modified food retail environment score					0.004 (−0.01, 0.02)	.53
Region size 4 (ref:1)					−0.01 (−0.05, 0.03)	.62
Region size 3 (ref:1)					0.01 (−0.06, 0.07)	.81
Region size 2 (ref:1)					0.00 (−0.05, 0.05)	.99
Store ownership, Sobeys (ref: Loblaws)					0.02 (−0.02, 0.05)	.44
Store ownership, Walmart (ref: Loblaws)					−0.03 (−0.07, 0.002)	.07
Mutli-unit package (ref: single unit)					−0.08 (−0.09, −0.07)	<.001

Estimates from autoregressive moving-average (ARMA) model fit with generalized least squares.

^a^‘Taxable’ refers to beverages that would be taxable under the NL SSB tax policy, if sold in NL, in any province.

### Total beverage sales

The retailer scanner data included annual sales of over 300 million L of ready-to-drink beverages sold across Atlantic Canada (NL + MAR) (pre-tax: 333 527 612 L; post-tax: 323 371 256 L), for total sales of $544 720 654 and $588 483 732 the year before and after the tax was implemented, respectively, unadjusted for inflation. SSBs (taxable and non-taxable) made up 42%–45% of the total volume of ready-to-drink beverages sold; SSBs that would be taxable under the NL policy made up 33%–38% of the total volume sold across all years and regions. Taxable SSBs—those that would be subject to the tax if sold in NL—made up the majority of the total volume of all SSBs sold (NL-pre-tax: 83.5%, NL-post-tax: 83.4%; MAR-pre-tax: 78.1%, MAR-post-tax: 78.9%). SSBs accounted for a decline in the proportion of total volume sold in NL (−3.0%pts; 45.1% vs 42.1%) whereas there was a slight increase in MAR (1.0%pts; 43.1% vs 42.1%) before and after the tax was implemented. The proportion of total volume taxable SSBs reduced by 2.5 percentage points in NL (pre-tax: 37.6% vs post-tax: 35.1%) and 0.4% in MAR (pre-tax: 33.6% vs post-tax: 33.2%) before and after the NL SSB tax was introduced.

#### Change in sales by beverage group per capita

**Figure 1.**
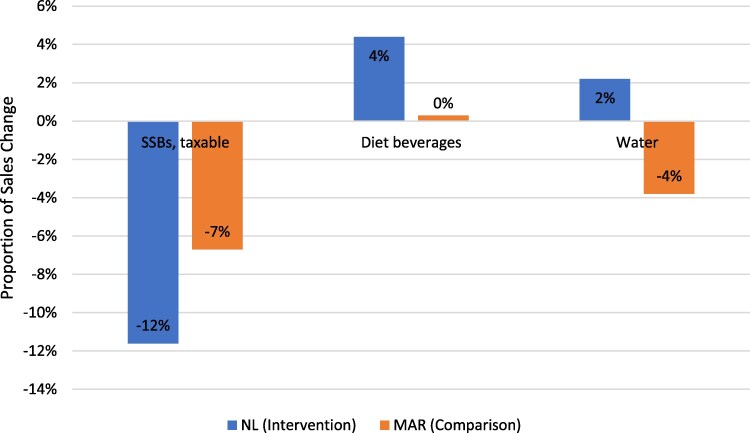
Change in sales of taxable SSBs, diet beverages, and water varieties in NL and the Maritime Provinces (MAR) between years before and after NL SSB tax implementation.

Between pre-tax and post-tax periods, per capita volume sold decreased for most beverage groups in MAR and in NL (Table 3). Per capita taxable SSB sales by volume decreased by 11.6% in NL, compared with a 6.7% decreased in MAR (Fig. 1). Diet beverages increased by 4.4% in NL but remained almost unchanged in MAR (0.3%). Per capita volume sold of all types of unsweetened water increased in NL (2.2%) but not MAR (−3.8%). On the other hand, per capita beverage sales in dollars increased for all groups in both regions, except for 100% fruit juice (−8.0% in NL and −8.5% in MAR) and chocolate milk (−0.2% in MAR) (Table 3). These changes represent a general overall increase in inflation-adjusted average prices per litre for all groups, except water in NL (−1.2%) and plain milk in MAR (−0.3%) (Table 3).

**Table 3.** Total beverage sales per 100 000 people in litres (l) and dollars (CAD) in Newfoundland and Labrador (intervention) and the Maritimes (control) 1 year before and after implementation of the NL SSB tax.

Beverage group	NL				MAR			
	Pre-tax^a^	Post-tax	Difference post-pre	% Change	Pre-tax^a^	Post-tax	Difference post-pre	% Change
SSBs, subject to the NL tax^b^								
Volume sold (l)	5 509 170	4 868 992	−640 178	−11.6	4 346 362	4 055 661	−290 700	−6.7
Dollars sold (CAD)	$7 367 421	$7 550 052	$182 631	2.5	$6 777 744	$7 230 339	$452 595	6.7
Average price per litre	$3.52	$3.68	$0.16	4.6	$3.63	$3.68	$0.05	1.5
Diet beverages								
Volume sold (l)	4 277 189	4 464 490	187 301	4.4	2 447 101	2 453 473	6373	0.3
Dollars sold (CAD)	$5 070 736	$6 071 418	$1 000 681	19.7	$3 423 549	$3 912 082	$488 532	14.3
Average price per litre	$2.65	$3.24	$0.59	22.3	$2.76	$3.27	$0.51	18.4
Chocolate milks								
Volume sold (l)	274 538	258 246	−16 291	−5.9	392 507	361 822	−30 684	−7.8
Dollars sold (CAD)	$698 478	$708 514	$10 036	1.4	$1 012 757	$1 010 419	−$2338	−0.2
Average price per litre^c^	NA	NA	NA	NA	NA	NA	NA	NA
100% juices								
Volume sold (l)	731 724	621 839	−109 885	−15.0	736 366	622 586	−113 780	−15.5
Dollars sold (CAD)	$1 630 692	$1 500 656	−$130 036	−8.0	$1 744 103	$1 596 578	−$147 524	−8.5
Average price per litre^c^	NA	NA	NA	NA	NA	NA	NA	NA
Plain milk								
Volume sold (l)	2 657 213	2 490 056	−167 157	−6.3	3 619 246	3 408 356	−210 890	−5.8
Dollars sold (CAD)	$5 492 649	$6 011 958	$519 308	9.5	$7 012 354	$7 200 128	$187 774	2.7
Average price per litre	$2.26	$2.41	$0.16	6.9	$2.12	$2.11	−$0.01	−0.3
Water								
Volume sold (l)	1 334 977	1 364 264	29 287	2.2	1 508 881	1 452 135	−56 746	−3.8
Dollars sold (CAD)	$1 216 994	$1 327 165	$110 172	9.1	$1 608 864	$1 662 914	$54 050	3.4
Average price per litre	$1.17	$1.16	−$0.01	−1.2	$1.16	$1.17	$0.00	0.4

^a^Adjusted for inflation.

^b^Refers to beverages that would be taxable under the NL SBB tax policy, if sold in NL, in any province.

^c^The volume and dollars sold of chocolate milks and 100% juices were calculated from beverage categories provided by ACNielsen. Sales of chocolate milk were calculated from sales of sugar-containing milk (e.g. chocolate + flavoured/shake milks) minus taxable milk (e.g. flavoured/shake milks). Sales of 100% juice were calculated from sales of juices and juice drinks with free/added sugars (e.g. 100%juice + fruit punch) minus taxable juices and juice drinks (e.g. fruit punch). As a result, the average price per litre for chocolate milk and 100% juice could not be accurately calculated.

#### Change in sales by beverage type per capita

The change in total volumetric sales by beverage category, categorized by sugar-containing versus non-sugar-containing, and taxable vs non-taxable (where taxable refers to beverages that would be subject to the tax if sold in NL), beverages for NL and MAR are presented in Supplemental File 2. Similar trends for changes in volume sold between pre-tax and post-tax periods exist for most beverage types between NL and MAR with the exception of the Juices and Drinks category (includes juices and juice-like fruit or vegetable beverages) which appeared to change more dramatically for NL compared with MAR [may be explained by changes in product availability in the market (personal communication, ACNielsen, 22 May 2024)].

In general, MAR had higher sales in litres per 100 000 people of most beverage types, except for soft drinks where NL had substantially higher per capita sales of regular soft drinks and diet soft drinks (Supplementary data). There was a greater decrease in regular soft drink sales in NL (−13.0%) compared with MAR (−7.6%), and a slight increase in diet soft drink sales NL (+1.3%), compared with a decrease in MAR (−3.2% in MAR).

NL also had higher per capita sales in litres for plain water but not flavoured water that was sugar-containing or non-sugar containing (Supplementary data). Plain water sales in litres increased in both regions (3.3% in NL; 2.4% in MAR), but the changes in flavoured water differed between regions. Non-sugar-containing flavoured water stayed constant in NL (0.1%), but decreased in MAR (−9.8%). Sugar-containing flavoured water sales increased in NL (3.4%) but decreased in MAR (−1.0%).

## DISCUSSION

This study examined changes in beverage prices and sales in NL, where Canada’s first provincial excise tax on SSBs was introduced, compared with non-tax regions between pre-tax and post-tax periods. Our analysis of online prices of 53 beverages from national grocers did not indicate a change in posted retail price of taxable SSBs in NL 3-months after the tax was introduced, compared with non-tax regions (NS, NB, MB, and YT). However, our analysis of market sales data for the year before and after the introduction of the NL SSB tax showed that average prices per litre have increased beyond inflation across broad beverage category types, coinciding with a general decrease in total litres of beverages sold. Sales of taxable SSBs decreased in both NL and non-tax regions (MAR) between study periods, but to a greater extent in NL. Regular sugar-sweetened soft drink sales decreased more, while diet soft drink sales increased more in NL compared with MAR. There was also a greater increase in plain water sales in NL compared with MAR, but no reduction in unsweetened flavoured water sales which was observed in MAR.

It is necessary to monitor the prices of beverages to assess pass-through of the tax—the proportion of the tax that consumers are required to pay versus the absorption of the tax by manufacturers, distributors, or retailers (WHO 2024). Monitoring tax pass-through to consumers in the form of increased prices is crucial, as the extent to which this occurs influences the extent to which the tax reduces demand of SSBs (Powell *et al*. 2021). In order for a tax on SSBs to be effective in reducing SSB consumption and promoting healthier beverage choices, the tax must raise the price of the targeted beverages for consumers. A variety of factors influence tax pass-through rates including market structure and price elasticity of demand; in markets that are competitive or display inelastic demand, pass-through is often higher in comparison to markets with elastic demand that tend towards only partial pass-through (Powell *et al*. 2021). Incomplete pass-through may reduce the potential impacts of the tax on consumer behaviour (i.e. buying fewer SSBs) which in turn impacts SSB consumption. When all or most of the tax amount is passed on to consumers, thus increasing prices for taxed beverages, SSB taxes can be expected to impact demand and support the public health goals of the intervention (Powell *et al*. 2021). This in turn may also influence consumer demand of non-taxable beverages. Monitoring of pass-through continuously after tax implementation is also vital for understanding potential changes (increases or decreases) in pass-through over time, which can continue to affect SSB sales and consumption (Powell *et al*. 2021). It has also been noted that communication of the tax on the ‘shelf’, or posted retail price, may be more influential to consumers when making purchasing decisions compared with taxes that are applied at point-of-sale, or checkout (Powell *et al*. 2021). The NL policy does not have requirements about where and how the tax is to be communicated to consumers, meaning the communication of the tax is decided by the stores themselves and is not required to be on the posted ‘shelf’ tag (Government of NL 2022).

Meta-analytic evidence suggests the average pass-through rate of SSB taxes is 82%, though this rate can vary widely—from negative values to over 100%—depending on the specific policy and location (Andreyeva *et al*. 2022). A tax pass-through rate of 82% would mean that on a $0.20/l SSB tax, only $0.16/l (82% of the cost of the tax) is being passed on to, or paid by, consumers who purchase taxed beverages. Pass-through rates often also vary by beverage and store type (El-Sayed *et al.* 2020, Leider et al, 2021). Berkeley, California, e.g. introduced a 1 cent per ounce excise tax on SSBs in 2014, applied at the level of the distributor. One year after its implementation, only 47% of the tax was passed through to the retail price of SSBs (Falbe *et al*. 2020), meaning beverage prices changed less than half of what was intended by the tax. The city provided little guidance to businesses on how to implement the tax, generally only engaging with self-distributors. Thus, individual retailers chose (i) how to pass the tax through to consumers and (ii) how to display the tax in store. The NL SSB tax requires registered wholesalers of SSBs to remit the tax to the government based on sales of SSBs to retailers and consumers (Government of NL 2022). Regulations appear to suggest that retailers are expected to transfer the surcharge to consumers at their own discretion; little guidance is provided to retailers besides that they must pay the tax to registered wholesalers and ‘levy and collect the sugar sweetened beverage tax from consumers’ (Government of NL 2022). They are not required to display the SSB tax on the customer’s receipt (Government of NL 2022).

Our research indicates that the NL SSB tax is under-shifted when assessed through online product pages before checkout. As a result, consumers did not encounter a price difference for SSBs subject to the tax when deciding to add an item to their online cart. This suggests that our findings may underestimate the full economic pass-through of the tax, as the NL SSB tax appears to be commonly applied at checkout rather than reflected in the displayed price. Notably, retailer scanner purchasing data, which reports in dollars, does not include the $0.20/L SSB tax (personal communication, ACNielsen, 29 February 2024), implying that the SSB tax is added at checkout as a sales tax or additional fee rather than being incorporated into the posted price. Other unpublished research from our team confirms that most retailers are applying the SSB tax at the register during checkout, where it appears in the tax line alongside other fees and taxes (e.g. carbon, environmental, electronic, or bottle fees), rather than affecting the posted price of the item directly. In other words, it is affecting the cost of the item, but not the price tag on shelves or on the product page online. This lack of change in the posted price at the point of decision-making reduces the likelihood that the SSB tax will act as a noticeable financial disincentive for purchasing SSBs.

Research consistently shows that taxes influence purchasing behaviour and dietary choices, as demonstrated by pre/post-intervention comparisons, experimental studies, and modelling studies (Jones *et al*. 2017, Acton *et al*. 2019, Eykelenboom *et al*. 2019, Powell and Leider 2020, Claudy *et al*. 2021). Therefore, incomplete tax pass-through and poor signalling to consumers could reduce the potential impact of an SSB tax. Powell and Leider (2020) examined the volume of beverages sold 1 year after SSB tax implementation in Seattle, finding the volume of taxed SSBs sold decreased by 22%, while the volume of untaxed beverages sold increased by 4%. A meta-analyses found SSB taxes significantly reduced SSB sales by 15% (95% CI −20% to −9%) (Andreyeva *et al*. 2022). With a 4.6% increase in taxable SSB price in NL after adjusting for inflation, we saw an associated decline of 11.6% in litres sold. There is a possible signalling effect of the SSB tax in NL, where consumers have altered behaviour because of their awareness of the tax despite the lack of visibility of the SSB tax at point of decision-making. Discrepancies between Study A (no significant price increase) and Study B (reported price increase; no statistical tests completed due to aggregate data) may be explained by the difference in the data collected. Implementation of the tax differs by beverage type and store type in other research (Cawley and Frisvold 2015). Study A included a set of indicator beverages monitored over time, and included data from online platforms for grocery, convenience, drug, and dollar stores. Study B included a comprehensive set of beverages in the market but was limited to grocery, mass merchandizers, and drug stores. The dataset used in Study B also captures point-of-sale data in physical stores, while Study A was limited to pre-checkout online data only.

In general, evidence demonstrates that SSB taxes are associated with lower sales, indicating a consumer response to economic interventions (WHO 2022). Our study suggests changes in beverage sales in NL compared with non-tax regions in ways generally expected following the implementation of an SSB tax (decreased sales of taxable SSBs, increased sales of non-taxable beverages, e.g. diet beverage, water). Substitution effects are important factors to consider when evaluating the impact of an SSB tax as the beverage replacement for an SSB will have implications on calorie intake and energy balance. Plain water is promoted as an ideal alternative beverage, according to Health Canada (Health Canada 2021) and the NL Government (Government of NL 2021); however, behaviour changes may not reflect recommendations. Our sales data suggests changes in the volume sold of sugar-sweetened and sugar-free flavoured water, which increased in NL compared with the decreases seen in MAR, though changes in plain water did not appear to greatly differ in NL compared with MAR. Future research is needed to understand the own-price elasticity of SSBs, cross-price elasticities of other beverages, and substitution effects in NL in response to the tax.

### Strengths and limitations

This is the first study to evaluate the impact of the NL SSB tax. As a natural experiment, it allows for real-life evaluation of an SSB tax; however, with lack of researcher control over the intervention, we encountered some challenges. Our study includes major retailers in Canada: collectively, Walmart, Sobeys, and Loblaws retailers make up 58% of the grocery market in Canada (Dalhousie University 2023). Our analysis of online prices is unique; however, it may not represent in-store pricing, or retailers without online platforms. Other research from our team has found no differences in the price of taxed SSBs and non-taxed beverages in-store in NL in the year before and after the tax (unpublished data). Our small sample of stores for beverage pricing is strengthened by the number of data collection periods pre-/post-tax which allowed us to use a controlled interrupted time series to evaluate the impact of the tax on beverage prices. With this study design, we can be confident in our conclusions that the NL SSB tax did not impact pre-checkout online beverage prices of 53 indicator beverages. Although our beverages sales dataset is extensive, including millions of sales across tax and non-tax regions, we cannot easily separate the effect of the tax from other factors that may impact purchasing. Further, we only were able to evaluate immediate effects of the tax over a 3-month period. With ongoing monitoring of beverage sales, future research will be more able to identify the direct impact of the SSB tax on purchasing. After the introduction of the SSB tax and our collection of pricing data, we realized that, across all store chains, the SSB tax was not visible on product selection pages; in some stores, it was added in a category of additional or other fees at checkout. Our pre-checkout data is important as it indicates the degree of communication of the SSB tax to consumers, but it may not accurately represent whether the consumer is paying all or any of the cost of the SSB tax. We noted that the SSB tax was not included in the retailer sale scanner data, which reinforces our pricing findings that retailers are not including the cost of the SSB tax in their ‘shelf price.

## CONCLUSION

NL faces significant challenges with diet and disease outcomes compared with other regions in the country. Taxing SSBs has been advocated by public health experts and researchers internationally, but NL is the first province to adopt such a fiscal public health intervention in Canada. Our study showed that the NL SSB tax had no immediate impact on retail prices of taxable SSBs which may reduce the potential impact of the policy on consumer behaviour change and warrants further exploration. Nevertheless, beverage purchasing patterns in NL have shifted since the implementation of the SSB tax. Although posted prices of beverages did not change, the cost of beverages is believed to have increased with the SSB tax commonly applied at check-out. Further research is needed to explore the full-pass through of the tax to consumers. This change aligns with anticipated outcomes of the SSB tax policy. It is, however, difficult to isolate the specific impact of the SSB tax from broader market trends or other influencing factors. The long-term effectiveness of the NL SSB tax will depend on its implementation and subsequent impacts. Rigorous studies are needed to identify factors related to tax design, retailer implementation, and consumer or industry responses that could either strengthen or weaken the ability of the SSB tax to improve the diets and health of Newfoundlanders and Labradorians.

## Supplementary Material

daaf203_Supplementary_Data

## Data Availability

Some data underlying this article were provided by AC Nielsen, for which the authors do not have permission to share data. The data that was collected by researchers during this study is not currently publicly available due to data privacy concerns. Requests for access to data can be made directly to the corresponding authors and will be evaluated on a case-by-case basis.
