# Supplementary material for: Evaluating sugar-sweetened beverage tax effects: online price and sales data from grocers in Canada
Source: Health Promot Int. 2025 Dec 2;40(6):daaf203. doi: 10.1093/heapro/daaf203 (PMC12669988; doi:10.1093/heapro/daaf203)
Supplement: daaf203_Supplementary_Data [file daaf203_supplementary_data.zip › SSB_Price Sales_online_supplemental file 1.docx]

**Supplemental File 1- Beverages Included in Beverage Tax Food Store Observation Form, adapted for Newfoundland and Labrador, Canada**

| **Product** | **Beverage Category** | **Subject to NL SSB Tax?** |
| --- | --- | --- |
| Coca-Cola Regular | Soft Drink | Yes |
| Diet Coke | Soft Drink | No |
| Sprite | Soft Drink | Yes |
| Sprite Zero Sugar | Soft Drink | No |
| Barq's Root Beer | Soft Drink | Yes |
| Pepsi Regular | Soft Drink | Yes |
| Diet Pepsi | Soft Drink | No |
| 7-UP | Soft Drink | Yes |
| 7-Up Zero Sugar | Soft Drink | No |
| Mountain Dew | Soft Drink | Yes |
| Canada Dry Ginger Ale | Soft Drink | Yes |
| Canada Dry Diet | Soft Drink | No |
| Canada Dry Club Soda | Soft Drink | No |
| Dr. Pepper | Soft Drink | Yes |
| Store brand Regular Cola | Soft Drink | Yes |
| Store brand Diet Cola | Soft Drink | No |
| Powerade | Sports Drink | Yes |
| Powerade Zero | Sports Drink | No |
| Gatorade | Sports Drink | Yes |
| Gatorade G2 (Low Sugar) | Sports Drink | Yes |
| Gatorade G Zero (No Sugar) | Sports Drink | No |
| Monster Energy | Energy Drink | Yes |
| Monster Energy (Zero Sugar) | Energy Drink | No |
| Monster Energy (Low Sugar) | Energy Drink | Yes |
| Red Bull Original | Energy Drink | Yes |
| Red Bull Sugar Free | Energy Drink | No |
| Minute Maid Orange | Juice Drinks | No |
| Tropicana Orange Juice | Juice Drinks | No |
| Tropicana Cranberry Cocktail | Juice Drinks | Yes |
| SunRype Apple Juice | Juice Drinks | No |
| 5 Alive Citrus | Juice Drinks | Yes |
| Kool-Aid Jammers | Juice Drinks | Yes |
| Arizona Green Tea | Lemonades and Iced Teas | Yes |
| Arizona Diet Green Tea | Lemonades and Iced Teas | No |
| Nestea Lemon Iced Tea | Lemonades and Iced Teas | Yes |
| Nestea Lemon Iced Tea Zero | Lemonades and Iced Teas | No |
| Brisk Iced Tea | Lemonades and Iced Teas | Yes |
| Pure Leaf Lemon Iced Tea | Lemonades and Iced Teas | Yes |
| Pure Leaf Lemon Iced Tea Zero | Lemonades and Iced Teas | No |
| Brisk Lemonade | Lemonades and Iced Teas | Yes |
| Minute Maid Lemonade | Lemonades and Iced Teas | Yes |
| Starbucks Vanilla Frappuccino | Coffee Drinks | Yes |
| Tim Hortons Iced Cappuccino | Coffee Drinks | Yes |
| Vitamin Water | Waters | Yes |
| Vitamin Water Zero Sugar | Waters | No |
| Bubly Water (Any Flavour) | Waters | No |
| AHA (Any Flavour) | Waters | No |
| Dasani Plain Water | Waters | No |
| Aquafina Plain Water | Waters | No |
| Scotsburn 2% Milk White  (Beatrice, Dairyland, Natre, Northumberland, Quebon in other provinces) | Milk and Soy Milk | No |
| Scotsburn 2% Milk Chocolate  (Beatrice, Dairyland, Natre, Northumberland in other provinces) | Milk and Soy Milk | No |
| Silk Soy Beverage Unsweetened | Milk and Soy Milk | No |
| Silk Soy Beverage Chocolate | Milk and Soy Milk | No |
