# Supplementary material for: Evaluating sugar-sweetened beverage tax effects: online price and sales data from grocers in Canada
Source: Health Promot Int. 2025 Dec 2;40(6):daaf203. doi: 10.1093/heapro/daaf203 (PMC12669988; doi:10.1093/heapro/daaf203)
Supplement: daaf203_Supplementary_Data [file daaf203_supplementary_data.zip › SSB_Price Sales_online_supplemental file 2.docx]

**Supplemental File 2 - Change in total volumetric sales by beverage category, categorized by sugar-containing versus non-sugar-containing, and taxable vs non-taxable, beverages for NL and MAR**

**Table 1: Total beverage sales (flavoured soft drinks, coffee type drinks, extreme energy drinks, RTD iced tea cans) per 100,000 people in litres (L) and dollars (CAD) in Newfoundland and Labrador (intervention) and the Maritimes (control) one year before and after the NL SSB tax was implemented.**

| Beverage Category | | NL | | | | MAR | | | |
| --- | --- | --- | --- | --- | --- | --- | --- | --- | --- |
|  |  | Pre-tax^1^ | Post-tax | Difference Post-Pre | % change | Pre-tax^1^ | Post-tax | Difference Post-Pre | % change |
| Flavoured soft drinks, sugar-sweetened^2^ | |  |  |  |  |  |  |  |  |
|  | Volume sold (L) | 4,095,434 | 3,564,074 | −531,360 | −13.0% | 2,866,528 | 2,647,933 | −218,595 | −7.6% |
|  | Dollars sold (CAD) | $4,561,440 | $4,665,560 | $104,120 | 2.3% | $3,545,939 | $3,813,358 | $267,419 | 7.5% |
|  | Average price per L | $1.22 | $1.31 | $0.09 | 7.5% | $1.35 | $1.44 | $0.09 | 6.5% |
| Flavoured soft drinks, unsweetened^3^ | |  |  |  |  |  |  |  |  |
|  | Volume sold (L) | 3,752,887 | 3,802,444 | 49,557 | 1.3% | 1,933,493 | 1,871,900 | −61,593 | −3.2% |
|  | Dollars sold (CAD) | $3,896,080 | $4,547,865 | $651,785 | 16.7% | $2,219,231 | $2,479,972 | $260,741 | 11.7% |
|  | Average price per L | $1.13 | $1.20 | $0.06 | 5.4% | $1.26 | $1.32 | $0.07 | 5.6% |
| Coffee type drinks, sugar-sweetened^2^ | |  |  |  |  |  |  |  |  |
|  | Volume sold (L) | 40,702 | 33,992 | −6,710 | −16.5% | 45,050 | 38,073 | −6,977 | −15.5% |
|  | Dollars sold (CAD) | $207,182 | $190,391 | −$16,791 | −8.1% | $232,272 | $220,773 | −$11,499 | −5.0% |
|  | Average price per L | $5.56 | $5.60 | $0.04 | 0.7% | $5.64 | $5.80 | $0.16 | 2.9% |
| Coffee type drinks, unsweetened^3^ | |  |  |  |  |  |  |  |  |
|  | Volume sold (L) | 3,456 | 3,989 | 533 | 15.4% | 4,735 | 5,289 | 554 | 11.7% |
|  | Dollars sold (CAD) | $16,597 | $21,528 | $4,931 | 29.7% | $22,569 | $28,816 | $6,248 | 27.7% |
|  | Average price per L | $5.25 | $5.40 | $0.15 | 2.8% | $5.21 | $5.45 | $0.24 | 4.5% |
| Extreme energy drinks, sugar-sweetened^2^ | |  |  |  |  |  |  |  |  |
|  | Volume sold (L) | 47,742 | 51,951 | 4,209 | 8.8% | 61,328 | 67,351 | 6,022 | 9.8% |
|  | Dollars sold (CAD) | $317,556 | $366,316 | $48,760 | 15.4% | $417,690 | $485,967 | $68,277 | 16.3% |
|  | Average price per L | $7.27 | $7.05 | −$0.22 | −3.0% | $7.45 | $7.22 | −$0.23 | −3.1% |
| Extreme energy drinks, unsweetened^3^ | |  |  |  |  |  |  |  |  |
|  | Volume sold (L) | 41,744 | 41,197 | −547 | −1.3% | 45,925 | 45,688 | −237 | −0.5% |
|  | Dollars sold (CAD) | $262,900 | $279,330 | $16,430 | 6.2% | $299,557 | $315,335 | $15,778 | 5.3% |
|  | Average price per L | $6.88 | $6.78 | −$0.10 | −1.5% | $7.13 | $6.90 | −$0.23 | −3.2% |
| RTD Iced tea cans, sugar-sweetened^2^ | |  |  |  |  |  |  |  |  |
|  | Volume sold (L) | 85,259 | 70,516 | −14,743 | −17.3% | 128,743 | 112,657 | −16,086 | −12.5% |
|  | Dollars sold (CAD) | $149,753 | $150,752 | $999 | 0.7% | $238,896 | $258,916 | $20,020 | 8.4% |
|  | Average price per L | $5.34 | $5.76 | $0.42 | 7.9% | $5.45 | $5.61 | $0.16 | 2.9% |
| RTD Iced tea cans, unsweetened^3^ | |  |  |  |  |  |  |  |  |
|  | Volume sold (L) | 15,130 | 14,950 | −180 | −1.2% | 23,655 | 24,480 | 826 | 3.5% |
|  | Dollars sold (CAD) | $36,471 | $42,167 | $5,696 | 15.6% | $70,599 | $79,792 | $9,193 | 13.0% |
|  | Average price per L | $1.32 | $1.41 | $0.09 | 7.1% | $1.63 | $1.63 | $0.00 | −0.1% |

^1^adjusted for inflation

^2^Sugar-sweetened beverages included here are also subject to the NL SSB Tax.

^3^Unsweetened beverages included here are those with no sugar added but may contain non-nutritive sweeteners or be diet varieties. These beverages are not subject to the NL SSB Tax.

**Table 2: Total beverage sales (juices and juice drinks) per 100,000 people in litres (L) and dollars (CAD) in Newfoundland and Labrador (intervention) and the Maritimes (control) one year before and after the NL SSB tax was implemented.**

| Beverage Category | | NL | | | | MAR | | | |
| --- | --- | --- | --- | --- | --- | --- | --- | --- | --- |
|  |  | Pre-tax^1^ | Post-tax | Difference Post-Pre | % change | Pre-tax^1^ | Post-tax | Difference Post-Pre | % change |
| Juice or juice drink, free/added sugars^2^ | |  |  |  |  |  |  |  |  |
|  | Volume sold (L) | 1,947,867 | 1,745,559 | −202,308 | −10.4% | 1,948,503 | 1,779,478 | −169,026 | −8.7% |
|  | Dollars sold (CAD) | $3,690,250 | $3,594,377 | −$95,873 | −2.6% | $3,988,199 | $3,813,358 | −$54,776 | −1.4% |
|  | Average price per L | $1.49 | $1.48 | −$0.01 | −0.5% | $1.60 | $1.60 | $0.00 | −0.1% |
| Juice or juice drink, taxable^3^ | |  |  |  |  |  |  |  |  |
|  | Volume sold (L) | 1,216,143 | 1,123,721 | −92,423 | −7.6% | 1,212,137 | 1,156,891 | −55,246 | −4.6% |
|  | Dollars sold (CAD) | $2,059,558 | $2,093,721 | $34,163 | 1.7% | $2,244,097 | $2,336,845 | $92,748 | 4.1% |
|  | Average price per L | $2.03 | $2.05 | $0.03 | 1.3% | $2.28 | $2.29 | $0.01 | 0.5% |
| Juice or juice drink, non-taxable^4^ | |  |  |  |  |  |  |  |  |
|  | Volume sold (L) | 963,245 | 920,569 | −42,677 | −4.4% | 954,954 | 872,546 | −82,408 | −8.6% |
|  | Dollars sold (CAD) | $2,059,240 | $2,080,590 | $21,349 | 1.0% | $2,148,179 | $2,087,508 | −$60,670 | −2.8% |
|  | Average price per L | $2.43 | $2.39 | −$0.04 | −1.8% | $2.54 | $2.51 | −$0.02 | −0.9% |

^1^adjusted for inflation

^2^ Juice or juice drink, free/added sugars includes 100% fruit and vegetable juices and all juice-like fruit or vegetable beverages with added sugars, including fruit juice concentrate.

^3^Juice or juice drink, taxable includes all juice-like fruit or vegetable beverages with added sugars, including fruit juice concentrate, except for beverages that were labelled 100% fruit juice. These are subject to the NL SSB Tax.

^4^Juice or juice drink, non-taxable includes 100% fruit and vegetable juices, and juice-like fruit or vegetable beverages with no added sugars (but may contain non-nutritive sweeteners). These are not subject to the NL SSB Tax.

**Table 3: Total beverage sales (milk and water) per 100,000 people in litres (L) and dollars (CAD) in Newfoundland and Labrador (intervention) and the Maritimes (control) one year before and after the NL SSB tax was implemented.**

| Beverage Category | | NL | | | | MAR | | | |
| --- | --- | --- | --- | --- | --- | --- | --- | --- | --- |
|  |  | Pre-tax^1^ | Post-tax | Difference Post-Pre | % change | Pre-tax^1^ | Post-tax | Difference Post-Pre | % change |
| Milk, sugar-sweetened | |  |  |  |  |  |  |  |  |
|  | Volume sold (L) | 274,692 | 258,442 | −16,249 | −5.9% | 396,432 | 366,230 | −30,202 | −7.6% |
|  | Dollars sold (CAD) | $699,155 | $709,543 | $10,388 | 1.5% | $1,030,036 | $1,032,194 | $2,158 | 0.2% |
|  | Average price per L | $2.78 | $2.75 | −$0.04 | −1.3% | $2.84 | $2.82 | −$0.02 | −0.8% |
| Milk, plain | |  |  |  |  |  |  |  |  |
|  | Volume sold (L) | 2,657,213 | 2,490,056 | −167,157 | −6.3% | 3,619,246 | 3,408,356 | −210,890 | −5.8% |
|  | Dollars sold (CAD) | $5,492,649 | $6,011,958 | $519,308 | 9.5% | $7,012,354 | $7,200,128 | $187,774 | 2.7% |
|  | Average price per L | $2.26 | $2.41 | $0.16 | 6.9% | $2.12 | $2.11 | −$0.01 | −0.3% |
| Milk, taxable^2^ | |  |  |  |  |  |  |  |  |
|  | Volume sold (L) | 154 | 196 | 42 | 27.2% | 3,925 | 4,407 | 482 | 12.3% |
|  | Dollars sold (CAD) | $676 | $1,028 | $352 | 52.1% | $17,279 | $21,776 | $4,496 | 26.0% |
|  | Average price per L | $4.79 | $5.24 | $0.45 | 9.3% | $4.81 | $4.94 | $0.13 | 2.6% |
| Milk, non-taxable^3^ | |  |  |  |  |  |  |  |  |
|  | Volume sold (L) | 2,931,751 | 2,748,302 | −183,449 | −6.3% | 4,011,753 | 3,770,179 | −241,574 | −6.0% |
|  | Dollars sold (CAD) | $6,191,128 | $6,720,472 | $529,344 | 8.6% | $8,025,110 | $8,210,547 | $185,436 | 2.3% |
|  | Average price per L | $2.31 | $2.45 | $0.14 | 5.9% | $2.19 | $2.18 | −$0.01 | −0.4% |
| Water, flavoured, sugar-sweetened | |  |  |  |  |  |  |  |  |
|  | Volume sold (L) | 23,735 | 24,543 | 808 | 3.4% | 28,650 | 28,349 | −301 | −1.0% |
|  | Dollars sold (CAD) | $71,256 | $82,284 | $11,028 | 15.5% | $81,572 | $92,706 | $11,134 | 13.6% |
|  | Average price per L | $2.59 | $2.85 | $0.27 | 10.3% | $2.58 | $2.64 | $0.06 | 2.4% |
| Water, flavoured, unsweetened^4^ | |  |  |  |  |  |  |  |  |
|  | Volume sold (L) | 443,843 | 444,076 | 234 | 0.1% | 763,165 | 688,364 | −74,802 | −9.8% |
|  | Dollars sold (CAD) | $688,343 | $748,767 | $60,424 | 8.8% | $1,083,973 | $1,080,369 | −$3,604 | −0.3% |
|  | Average price per L | $1.70 | $1.69 | −$0.01 | −0.5% | $1.55 | $1.57 | $0.02 | 1.1% |
| Water, plain^5^ | |  |  |  |  |  |  |  |  |
|  | Volume sold (L) | 891,134 | 920,187 | 29,053 | 3.3% | 745,716 | 763,771 | 18,055 | 2.4% |
|  | Dollars sold (CAD) | $528,651 | $578,398 | $49,748 | 9.4% | $524,891 | $582,545 | $57,654 | 11.0% |
|  | Average price per L | $0.65 | $0.63 | −$0.02 | −3.1% | $0.77 | $0.76 | −$0.01 | −0.9% |

^1^adjusted for inflation

^2^Milk, taxable includes all sugar-sweetened milk except for chocolate milk which was explicitly excluded from being subject to the NL SSB Tax. However, all chocolate ‘shake’-based milks are subject to the tax.

^3^Milk, not taxable includes all plain milk and chocolate milk (except ‘shake’-based milks) and are not subject to the NL SSB Tax.

^4^Water, unsweetened includes water with no sugar added but may contain non-nutritive sweeteners or be a diet variety of flavoured water. These beverages are not subject to the NL SSB Tax.

^5^Water, plain includes water with no added sweeteners (sugars or non-nutritive). May be carbonated or not, flavoured or not. These beverages are not subject to the NL SSB Tax.
